# Biomechanical investigation of the hybrid modified cortical bone screw–pedicle screw fixation technique: Finite-element analysis

**DOI:** 10.3389/fsurg.2022.911742

**Published:** 2022-07-18

**Authors:** Alafate Kahaer, Xieraili Maimaiti, Julaiti Maitirouzi, Shuiquan Wang, Wenjie Shi, Nueraihemaiti Abuduwaili, Zhihao Zhou, Dongshan Liu, Abulikemu Maimaiti, Paerhati Rexiti

**Affiliations:** ^1^ Department of Spine Surgery, The First Affiliated Hospital of Xinjiang Medical University, Urumqi, China; ^2^ College of Mechanical Engineering, Xinjiang University, Urumqi, China; ^3^ Department of Anatomy, College of Basic Medicine, Xinjiang Medical University, Urumqi, China; ^4^ First Clinical Medical College, Xinjiang Medical University, Urumqi, China; ^5^ Department of Imaging Center, The First Affiliated Hospital of Xinjiang Medical University, Urumqi, China

**Keywords:** traditional trajectory, modified cortical bone trajectory, hybrid fixation technique, 3-dimensional finite-element analysis, lumbar spine

## Abstract

**Background:**

Hybrid fixation techniques including the both modified cortical bone trajectory (MCBT) and traditional trajectory (TT) at the L4 and L5 lumbar segment are firstly proposed by our team. Therefore, the purpose of this study is to evaluate and provide specific biomechanical data of the hybrid fixation techniques including the MCBT and TT.

**Methods:**

Four human cadaveric specimens were from the anatomy laboratory of Xinjiang Medical University. Four finite-element (FE) models of the L4–L5 lumbar spine were generated. For each of them, four implanted models with the following fixations were established: TT-TT (TT screw at the cranial and caudal level), MCBT-MCBT (MCBT screw at the cranial and caudal level), hybrid MCBT-TT (MCBT screw at the cranial level and TT screw at the caudal level), and TT-MCBT (TT screw at the cranial level and MCBT screw at the caudal level). A 400-N compressive load with 7.5 N/m moments was applied to simulate flexion, extension, lateral bending, and rotation, respectively. The range of motion (ROM) of the L4–L5 segment and the posterior fixation, the von Mises stress of the intervertebral disc, and the posterior fixation were compared.

**Results:**

Compared to the TT-TT group, the MCBT-TT showed a significant lower ROM of the L4–L5 segment (*p* ≤ 0.009), lower ROM of the posterior fixation (*p* < 0.001), lower intervertebral disc stress (*p* < 0.001), and lower posterior fixation stress (*p *≤ 0.041). TT-MCBT groups showed a significant lower ROM of the L4–L5 segment (*p* ≤ 0.012), lower ROM of the posterior fixation (*p* < 0.001), lower intervertebral disc stress (*p < *0.001), and lower posterior fixation stress (*p* ≤ 0.038).

**Conclusions:**

The biomechanical properties of the hybrid MCBT-TT and TT-MCBT techniques at the L4–L5 segment are superior to that of stability MCBT-MCBT and TT-TT techniques, and feasibility needs further cadaveric study to verify.

## Introduction

For decades, the pedicle screw fixation technique has been the mainstay in the lumbar spine surgery (1), but it has several defects such as screw loosening, breakage, and extensive muscle dissection (2, 3), these were more common in patients with osteoporosis (4, 5). To acquire superior fixation strength, scholars had made many attempts from the shape design of screw to the curing of screw tracks, such as designing expandable pedicle screw, hydroxyapatite coating of the screw surface, and cement augmentation (6, 7). However, they have not been popularized in the clinic due to the high price, potential safety hazards including cement polymerization fever, chemical toxicity, and leakage.

**Figure 1:**
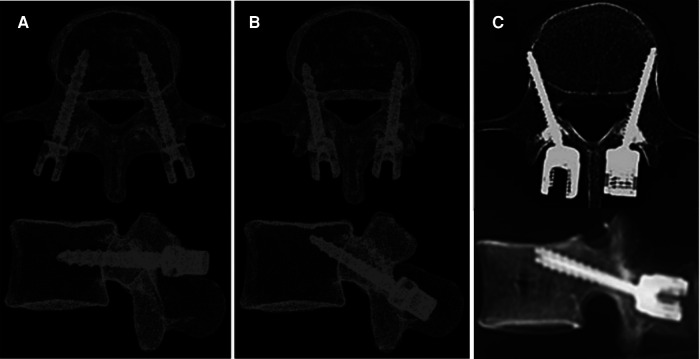
Schematic diagram of three different lumbar posterior fixations: (**A**). traditional trajectory (TT) (9), (**B**) cortical bone trajectory (CBT) (9), and (**C**) modified cortical bone trajectory (MCBT) (12).

In 2009, Santoni et al. (8) proposed the cortical bone trajectory (CBT) technique, changing the long axis of cancellous bone as a screw path compared to the pedicle screw. Cortical bone, on the other hand, does not produce significant osteopenia with degeneration compared with cancellous bone. The trajectory was 10 deg laterally in the axial plane and 25 deg cranially in the sagittal plane (9) (Figure 1B), with the entry points located at the 5 and 7 o’clock orientation of the left and right pedicle, respectively (10). CBT technique increased the pullout load by 30%, torque by 1.7 times (8, 11). However, cortical bone throughout the screw track has not been unreservedly used by CBT, especially the medial wall of the pedicle and the lateral margin of the superior endplate (12). Therefore, to compensate the defects in anatomical reference, entry point, and screw trajectory, we previously proposed the modified cortical bone trajectory (MCBT) technique (12) (Figure 1C). The insertion point of the MCBT technique close to the midline increasing the purchase of the screw because of the contact between the screw and the medial wall of the pedicle (12). MCBT technique avoids the facet joint degeneration caused by the interaction between the facet joint and the screw hub (13). In addition, increasing of the medio-lateral angle and screw length makes the screw head contact with the cortical bone in the anterior lateral edge of the upper endplate of the vertebral body. The MCBT technique makes full use of the cortical bone of inferior wall of pedicle by reducing cranio-caudal angle, especially in transforaminal lumbar interbody fusion (TLIF) (13). This was consistent with the results of Petrone et al. (14), Penner et al. (15), and Marengo et al. (16). Decompression of the lateral recess on the caudal vertebral body is often required, while complete decompression of the lateral recess cannot be completed after the MCBT was used. In addition, complete decompression may destroy the insertion point of the CBT and the MCBT screws. Isthmic fracture or lumbar spondylolisthesis was a contraindication for CBT and MCBT techniques (8, 9). Calvert et al. (17) demonstrated that CBT could be an alternative when the failure of the pedicle screw fixation occurs.

We firstly proposed the hybrid MCBT-TT and TT-MCBT techniques to make up for the defects of CBT and MCBT techniques, especially nerve decompression effect, and aim to provide the fixation technique with superior stability and strength. The ROM of the fixation segment and posterior fixation are inversely proportional to the stability of the vertebral body and the fixation. The equivalent stress of the intervertebral disc and the fixation are related to stability of the cage after lumbar fusion and the strength limit of fixation (18). In this study, the biomechanical properties of the hybrid techniques were discussed in detail by FE analysis method.

## Materials and methods

### Model development of the L4–L5 lumber spine

We have completed the construction and validation of the FE models in L4–L5 lumbar spine and analyzed the TT-TT group in the earlier study (19). In this study, the construction methods, material properties, and meshes were referred to the previous models (19). The cortical bone was defined as the 0.5 mm thickness outward from the outer layer of cancellous bone according to the density mapping based on CT scan data (20). Intervertebral disc was composed of incompressible annulus fibrosus and nucleus pulposus. The fluid-like behavior of the incompressible nucleus pulposus was simulated with linear elasticity (Poisson's value = 0.45). Meshed models were finally generated with a maximum size of 1.5 mm and a minimum size of 0.5 mm. ABAQUS 2019 (ABAQUS, Providence, Rhode Island, USA) was used for the material properties setting and FE analysis.

### Construction of surgical models

**Figure 2:**
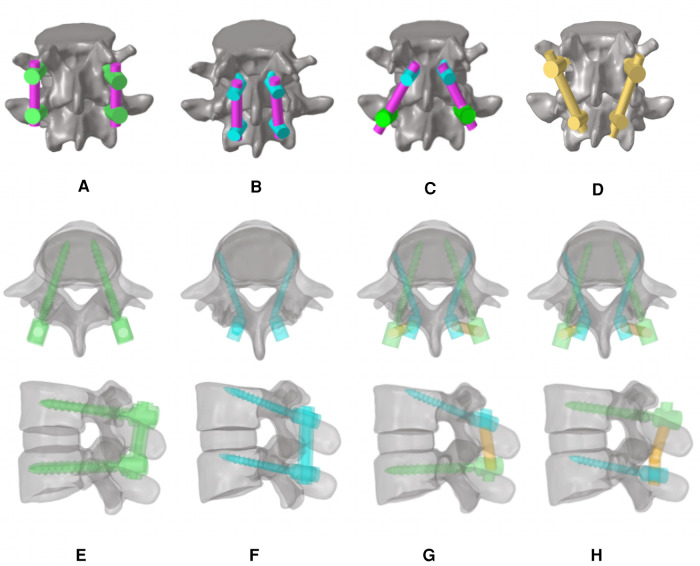
FE models of the lumbar vertebra and diagrams from the axial and sagittal views: (**A**) TT screws at the cranial and caudal levels (TT-TT) (19), (**B**) MCBT screws at the cranial and caudal levels (MCBT-MCBT), (**C**) MCBT screws at the cranial level and TT screws at the caudal level (MCBT-TT), (**D**) TT screws at the cranial level and MCBT screws at the caudal level (TT-MCBT), and (**E(19)–H**) were the axial and sagittal views of each respective technique of (**A–D**).

**Figure 3:**
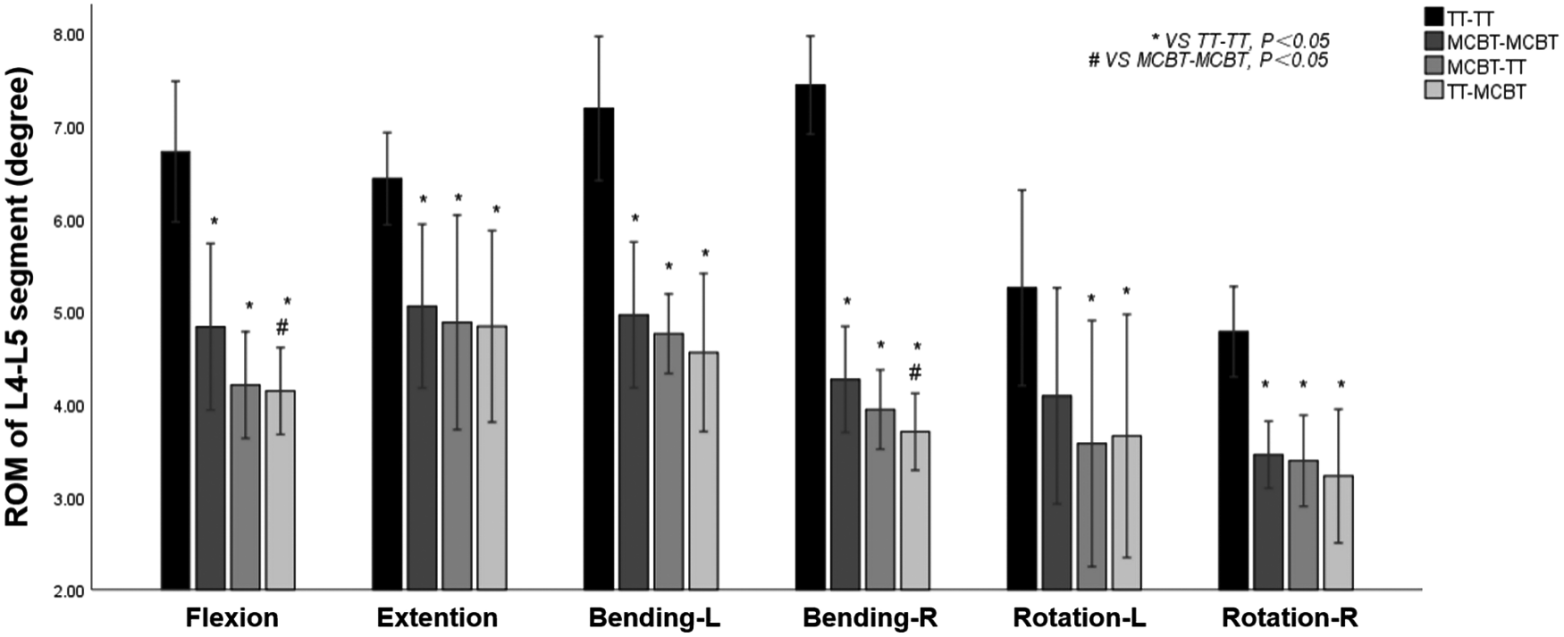
Mean values and minimum–maximum ranges of the ROMs of the fixation segment in four fixation models.

Four different posterior fixations were established: (1) TT-TT group, pedicle screws at the cranial, and caudal levels (Figures 2A,E) (19); (2) MCBT-MCBT group, MCBT screws at the cranial, and caudal levels (Figures 2B,F); (3) MCBT-TT group, MCBT screws at the cranial level, and TT screws at the caudal level (Figures 2C,G); and (4) TT-MCBT group, TT screws at the cranial level, and MCBT screws at the caudal level (Figures 2D,H). Sixteen FE models were established based on the four specimens. The insertion point of the MCBT screw was demonstrated in the earlier studies (12, 13) (Figures 2B,F). The TT screw is 6.0 mm in diameter and 45 mm in length, the MCBT screw is 4.5 mm in diameter and 40 mm in length, and titanium-based rod measured 5.5-mm in diameter.

### Boundary and loading conditions

All models were fixed at the inferior surface of the L5 vertebral body. A 400 N vertical axial preload with 7.5 Nm moment was applied on the superior surface of the L4 vertebral body to simulate flexion, extension, left-right lateral bending, and left-right rotation. The relationship between the L4 and L5 vertebral body and the intervertebral disc was defined as mutual contact, using face-to-face correspondence. The ROM of the L4–L5 segment and posterior fixation, von Mises stress of the intervertebral disc, and posterior fixation were investigated.

### Statistical methods

SPSS 26.0 software was used for data analysis and processing. Mean values of quantitative data were presented as mean ± standard deviation. One-way analysis of variance was used for the analysis of differences. When the differences were statistically significant, *post hoc* test was performed using the least significant difference (LSD) method. For all results, *p* < 0.05 was considered statistically significant.

## Results

### ROM of the L4–L5 segment

Compared to the TT-TT group, the MCBT-TT group showed a significantly lower ROM (*p* ≤ 0.012), and compared with the MCBT-MCBT group, the TT-MCBT group showed significantly lower ROM only in flexion and right lateral bending (*p* ≤ 0.045). There was no significant difference between the MCBT-TT group and the TT-MCBT group (*p* > 0.05). Among the four posterior fixation models, the TT-MCBT group showed the lowest ROM (Figure 3). Compared to the TT-TT group, ROM of the MCBT-TT group reduced by 37%, 24%, 34%, 47%, 32%, and 29% in flexion, extension, left-right bending, and left-right rotation, respectively. The TT-MCBT group reduced by 38%, 25%, 37%, 50%, 30%, and 33% in all working conditions, respectively. Compared to the MCBT-MCBT group, the TT-MCBT reduced by 14% and 13% in flexion and right lateral bending, respectively.

### ROM of the posterior fixation

**Figure 4:**
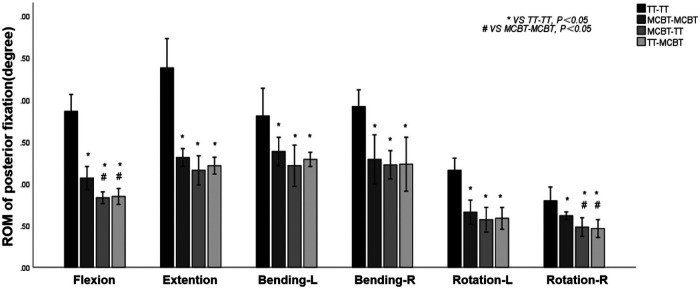
Mean values and minimum–maximum ranges of the ROMs of posterior fixations in four fixation models.

Compared to the TT-TT group, the MCBT-TT, and the TT-MCBT group showed significantly lower ROM in all working conditions (*p* < 0.001). Compared to the MCBT-MCBT group, the MCBT-TT group showed significantly lower ROM only in flexion and right rotation conditions (*p* ≤ 0.021). There was no significant difference between the MCBT-TT and TT-MCBT groups in all working conditions (*p* > 0.05). Among the four posterior fixation models, the TT-TT group showed the largest ROM (2.38° ± 0.22°) and the TT-MCBT group showed the lowest ROM (0.46° ± 0.07°) (Figure 4). Compared to the TT-TT group, MCBT-TT group reduced by 55%, 51%, 33%, 36%, 51%, and 39% in flexion, extension, left-right lateral bending, and left-right rotation, respectively. The TT-MCBT group was reduced by 55%, 49%, 29%, 36%, 49%, and 42% in all conditions, respectively. Compared to the MCBT-MCBT group, MCBT-TT was reduced by 13% and 2% in flexion and right rotation, respectively. The TT-MCBT was reduced by 15% and 7% in flexion and right rotation, respectively.

### Von Mises stress of the intervertebral disc

**Figure 5:**
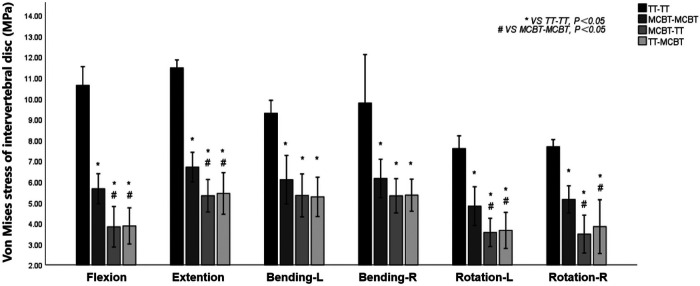
Mean values and minimum–maximum ranges of disc stresses at the fixation segment in four fixation models.

Compared to the TT-TT group, the MCBT-MCBT, MCBT-TT, and TT-MCBT groups showed significantly lower disc stress (*p* < 0.001). Compared to the MCBT-MCBT group, the MCBT-TT group was significantly reduced in flexion, extension, and rotation (*p* < 0.006), and there was no significant difference in lateral bending (*p* > 0.05). There was no significant difference between the MCBT-TT and TT-MCBT groups (*p* > 0.05). Compared to the TT-TT group, MCBT-MCBT group was reduced by 47%, 42%, 34%, 37%, 37%, and 33% in flexion, extension, left-right lateral bending, and left-right rotation, respectively. These were 64%, 54%, 42%, 46%, 53%, and 55% in the MCBT-TT group, and 64%, 53%, 43%, 45%, 52%, and 50% in the TT-MCBT group. Compared to the MCBT-MCBT group, MCBT-TT group was reduced by 32%, 21%, 26%, and 32% in flexion, extension, left rotation, and right rotation, respectively. These were 32%, 19%, 24%, and 25%, respectively in the same working conditions, in the TT-MCBT group (Figure 5).

### Von Mises stress of the posterior fixation

**Figure 6:**
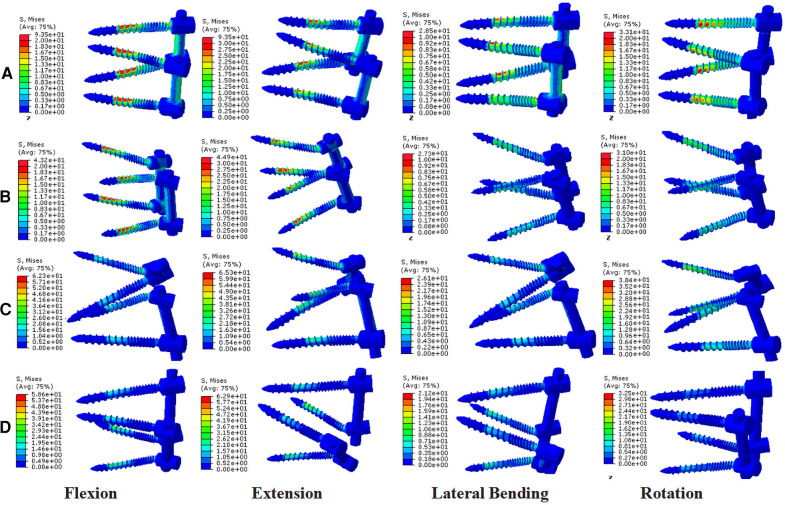
Stress nephograms over the posterior fixations of four different fixation models: (**A**) the TT-TT group, (**B**) the MCBT-MCBT group, (**C**) the MCBT-TT group, and (**D**) the TT-MCBT group.

**Figure 7:**
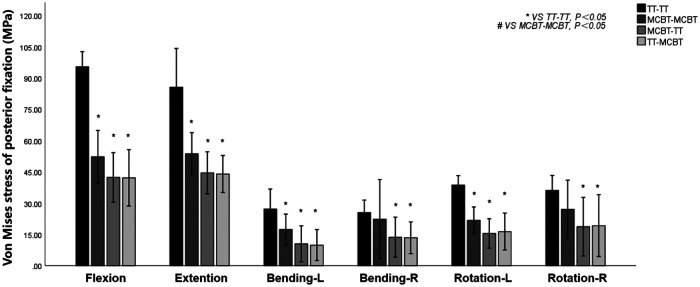
Mean values and minimum–maximum ranges of the posterior fixation stress in four fixation models.

The MCBT-TT and TT-MCBT groups showed superior load-sharing ability, and the von Mises stress of the posterior fixations were lower than that of the TT-TT and MCBT-MCBT groups (Figure 6). Compared to the TT-TT group, the posterior fixation stress was significantly lower in the MCBT-MCBT group in flexion, extension, left lateral bending, and left rotation conditions (*p* < 0.021), and significantly lower in the MCBT-TT and TT-MCBT groups in all conditions (*p* < 0.041). There was no significant difference between the MCBT-TT and MCBT-MCBT groups (*p* > 0.05). Compared to the TT-TT group, MCBT-MCBT group was reduced by 45%, 37%, 36%, and 44% in flexion, extension, left lateral bending, and left rotation, respectively. In the MCBT-TT group, the posterior fixation stress was reduced by 56%, 48%, 62%, 47%, 60%, and 48% in flexion, extension, left-right lateral bending, and left-right rotation, and 56%, 49%, 64%, 48%, 58%, and 47% in the TT-MCBT group, respectively (Figure 7).

## Discussion

The MCBT technique was previously proposed by our team (12, 13), aiming to make full use of the cortical bone, especially the medial wall of the pedicle and the lateral cortical bone of the superior endplate of the vertebral body. Reviewing the anatomy of the lumbar spine, the thickness of the bony cortex around the pedicle varies as follows: inferior wall > superior wall > medial wall > lateral wall (21). Fujiwara et al. (22) demonstrated that insertional torque increases with the increase of the medio-lateral angle and decrease of the distance from the anterior lateral edge of the upper endplate of the vertebral body. The screw insertion point of the MCBT technique moved 2.0–3.0 mm inward based on CBT, increased the bony cortex around the screw insertion point and medial wall of the pedicle and the anterior lateral edge of the upper endplate of the vertebral body by increasing the medio-lateral angle from 10° and decreasing the cranio-caudal angle from 25° according to the CBT technique, and it further increased the insertional torque (12, 13, 22). Petrone et al. (14) reached the superior anterior third of the vertebral endplate by decreasing the both cranio-caudal and medio-lateral angle of the screw to improve the biomechanical properties. Edwards et al. (23) demonstrated that cortical bone of the endplate in the marginal zone was thicker. Apparently, the holding force of the screw increases with the content of the thicker cortical bone around the screw. If the entry point moves inward, it can get the effect of contacting the medial wall of the pedicle, extending the screw length, and reaching the anterior lateral edge of the upper endplate of the vertebral body. In a nutshell, whether to increase or decrease the medio-lateral angle of the screw need further investigation with larger sample size. MCBT technique compensated the defects of the CBT technique. However, we found that the MCBT technique still has certain defects. Complete decompression of the recess was not possible with MCBT screw fixation, because extended decompression may destroy the insertion point of the MCBT screw. Isthmic fracture was also a contraindication to the MCBT technique. In summary, we proposed the hybrid screw insertion technique of MCBT-TT and TT-MCBT to compensate the defects of CBT or MCBT techniques. In a nutshell, this study confirms the *triangle stable effect* (24, 25) once again that stability of the hybrid MCBT-TT and TT-MCBT techniques is superior to the counterparts which had higher parallelogram-effect (Figures 2G,H).

Santoni et al. (8) and Matsukawa et al. (26) demonstrated with *in vitro* experiment that CBT screw has a higher purchase compared to the pedicle screw. However, Baluch et al. (27), demonstrated that there was no significant difference in the pullout strength. The differences in experimental results may be due to the deviation of the insertion point and angle, which causes the bias. The destruction of the screw trajectory causes an inability to insert the screw twice in a cadaveric study. However, FE analysis can replicate the model, and with the premise of 3D reconstruction of the lumbar spine, complete, accurate, and idealized screw insertion can be performed, which ensures the accuracy and reproducibility of the experiment and avoids the interference of confounding factors on the experimental results.

Matsukawa et al. (26) demonstrated that, compared to the TT-TT group, the CBT-CBT group showed superior vertebral stability in flexion and extension, while inferior in lateral bending. In the present study, compared to the TT-TT group, the ROM of the fixation segment in the MCBT-TT group was significantly reduced in all working conditions (Figure 3) (*p* ≤ 0.012). This difference may be due to the length of the MCBT screw (*L* = 40 mm), the head and hub of the MCBT screw were in the cortical bone area while the CBT screw hub was in the cortical bone, but the head is not. McLachlin et al. (28) proposed the *teeter-totter* mechanism, that is, the increase in the ROM of the screw caused by the swing of the screw head while the screw hub was fixed. However, both head and hub of the MCBT screw were in the cortical bone, it was not prone to swing. There was no significant difference between the MCBT-TT group and the TT-MCBT group (*p* > 0.05). In patients with osteoporosis, the purchase of the screw was not strong because of the reduced bone mineral density in cancellous bone (29), which led to the occurrence of the *teeter-totter* phenomenon. However, the *teeter-totter* phenomenon was not easy to occur when the head of the MCBT screw reaches the cortical bone at the lateral cortical bone of the superior endplate. Matsukawa et al. (30) demonstrated that the screw with greater length improves the fixation strength in rotation. The length of pedicle screw used in this study was 45 mm and the MCBT screw (*L* = 40 mm), while the pedicle screw diameter (*D* = 6.0 mm) was thicker than the MCBT screw (*D* = 4.5 mm). Although the length of the two screws were similar, but the ROM of the posterior fixations were different because the bony environment at the head and hub of the two screws were different. These results indicated that screw thickness or length was not an independent factor determining the stability of the fixation, which was consistent with the results of McLachlin et al. (28). In the TT-TT group, the intervertebral disc stress was more concentrated and higher, with the greatest stress in extension (11.47 ± 0.24 Mpa). In the MCBT-TT group, the intervertebral disc stress was more dispersed and lower, with the least stress in rotation (3.48 ± 0.57 Mpa). Rastegar et al. (31) demonstrated that cage subsidence was one of the risk factors for failure of lumbar interbody fusion. With the increase of cage stress, subsidence rate increased gradually (18). However, the lumbar fusion was not performed in this study, but it can be deduced from the intervertebral disc stress that lumbar interbody fusion combined with hybrid fixation techniques may reduce the cage stress and occurrence of cage subsidence increasing the bone fusion rate after lumbar fusion surgery. Von Mises stress of the pedicle screw in the TT-TT group evenly dispersed between the central cancellous bone area and screw hub, and the stress value was higher but evenly dispersed (Figure 6), which was consistent with the previous study of Chen et al. (32). Von Mises stress of the pedicle screw was significantly reduced in the MCBT-TT and the TT-MCBT groups. The stress of the pedicle screw in the TT-TT group was the largest in flexion condition (95.45 ± 4.55 Mpa), which was significantly reduced in the MCBT-TT group (42.35 ± 7.46 Mpa) and the TT-MCBT group (42.13 ± 8.50 Mpa) (*p* < 0.05), both of them were reduced by 56%. This result was consistent with the results of Ren et al. (33). Hybrid techniques have a superior stress dispersion effect, which may reduce the risk of fixation failure. Wang et al. (34) simulated the thoracolumbar FE model and demonstrated that the von Mises stress of the CBT-TT group was higher in lateral bending but lower in the other conditions compared to the TT-TT group during cross-stage fixation. In the present study, the von Mises stress of the MCBT-TT group was lower in lateral bending compared to the TT-TT group, although the diameter of the CBT screw (*D* = 5.0 mm, *L* = 35 mm) in the study of Wang et al. (34) was thicker than the MCBT screw (*D* = 4.5 mm, *L* = 40 mm) used in this study, but the length was shorter than the MCBT screw. The possible reason for this difference was that the contact of the screw head with the anterior lateral edge of the upper endplate of the vertebral body, the increase in screw length improved the stability of the vertebral body, which was consistent with the results of Matsukawa et al. (10).

Polly et al. (35) demonstrated that torque was reduced by 34% when the pedicle screw was re-placing after it was pulled out. Increasing the screw length and diameter when re-placing the screw improved the fixation strength, when screw diameter increases by 1 mm and the screw length increases by 5–10 mm, the fixation strength of the re-placed screw was similar to that of the original screw, but there was a risk of pedicle burst. Matsukawa et al. (30) demonstrated that pedicle screw with a larger diameter and longer length significantly improved the pullout strength, and the screw head reached the anterior lateral edge of the upper endplate of the vertebral body of the vertebral body to form a structure similar to bi-cortical fixation. The MCBT screw used in the present study had a smaller diameter (*D* = 4.5 mm), MCBT screw with a larger diameter may further increase the stability of hybrid fixation. Pedicle screw damages the facet joints inevitably, resulting in reduced vertebral stability and adjacent segment degeneration (ASD) (36), MCBT screw can avoid this problem. Masaki et al. (37) and Rodriguez et al. (38) demonstrated that CBT screw can be used cranially when ASD occurs at the proximal level and the fusion stage needed to be extended after lumbar surgery with the pedicle screw. The results of the present study were consistent with the above results. If a revision surgery was required for postoperative ASD, the MCBT screw can be an alternative for the pedicle screw. The non-parallel trajectory of pedicle screws in the same vertebral body had the *triangle stable effect* (24, 25), which increases the stability of posterior fixation. The results of the present study demonstrated that the stability of the hybrid MCBT-TT and the TT-MCBT techniques were superior to that of the TT-TT and the MCBT-MCBT techniques with the contribution of the *triangle stable effect* (24, 25).

As with any FE analysis, certain limitations were inherent in the present study. First, there was a lack of an adjacent segment that provides an indispensable role in evaluating the relationship between hybrid techniques and ASD. Second, the limited number of specimens was insufficient to allow more convincing conclusions. Third, there was only one size of TT and MCBT screws, thus the biomechanical effects of various screw diameters and lengths in the hybrid techniques have not been considered extensively in the present study. The present study did not standardize the sizes of the MCBT and the TT screws in the hybrid techniques.

## Conclusion

This study reflects the biomechanical properties of the hybrid MCBT-TT and TT-MCBT techniques. The stability of the hybrid MCBT-TT and the TT-MCBT techniques were superior to that of TT-TT and MCBT-MCBT techniques, providing a preliminary biomechanical theoretical basis for the application of hybrid lumbar fixation techniques in patients with osteoporosis. However, the present study did not analyzed the effects of the hybrid techniques on the adjacent segment. The authors concluded that the hybrid MCBT-TT and the TT-MCBT techniques can be the optimal choices for lumbar posterior fixation in patients with osteoporosis.

## Data Availability

The raw data supporting the conclusions of this article will be made available by the authors, without undue reservation.
